# Connaissances et pratiques des mères en matière d’alimentation des enfants de 0 à 24 mois dans la sous-préfecture de Sibut, République centrafricaine en 2022

**DOI:** 10.11604/pamj.2025.52.6.48021

**Published:** 2025-09-04

**Authors:** Ange Donatien Ngouyombo, Sylvain Woromogo, Stéphanie Ines Garoua-Adjou, Christelle Corinne Folefack Tatsadong, Rodrigue Herman Doyama-Woza, Jean de Dieu Longo

**Affiliations:** 1 Centre Inter-Etats d'Enseignement Supérieur en Santé Publique d'Afrique Centrale, Brazzaville, Congo,; 2 École Doctorale des Sciences Humaines et Vétérinaires, Université de Bangui, Bangui, République Centrafricaine,; 3 Ministère de la Santé et de la Population, Bangui, République Centrafricaine,; 4 Département de Santé Publique, Faculté des Sciences de la Santé, Université de Bangui, Bangui, République Centrafricaine

**Keywords:** Connaissance, pratique, alimentation, enfant, République centrafricaine

## Aux éditeurs de Pan African Medical Journal

La malnutrition est une condition pathologique résultant des déséquilibres dans l'apport énergétique et/ou nutritionnel d'une personne [1]. Dans les pays en développement, environ 5,4 millions d'enfants de moins de cinq ans meurent chaque année principalement des maladies ayant des causes évitables [2]. L'Organisation mondiale de la Santé (OMS) estime à environ 2,7 millions le nombre annuel de décès d'enfants imputables à la sous-nutrition, soit 45% de tous les décès d'enfants [3]. Des facteurs individuels, familiaux, communautaires, nationaux et internationaux allant de la maladie, des croyances culturelles et des coutumes, aux faibles conditions économiques et à l'accès limité aux services de santé et sociaux interviennent dans la survenue de la malnutrition. Elle entraîne des séquelles comme l'infirmité, la vulnérabilité chronique aux infections et une baisse des capacités intellectuelles [4].

En République centrafricaine (RCA), les résultats de l'enquête nutritionnelle SMART 2019 révèlent une prévalence nationale de la malnutrition aiguë globale (MAG) de 5,8%, classée comme moyenne selon les critères de l'OMS, et une prévalence de la malnutrition chronique globale (MCG) de 42,3%, indiquant une situation préoccupante pour le développement des enfants [5]. L'amélioration des pratiques d'alimentation du nourrisson et du jeune enfant (ANJE) constitue une stratégie clé pour prévenir la malnutrition. L'OMS recommande notamment l'allaitement maternel exclusif pendant les six premiers mois, suivi de l'introduction d'aliments complémentaires appropriés [6,7]. Malgré l'existence de directives nationales en RCA, les données sur les connaissances et pratiques des mères restent limitées.

**Figure 1:**
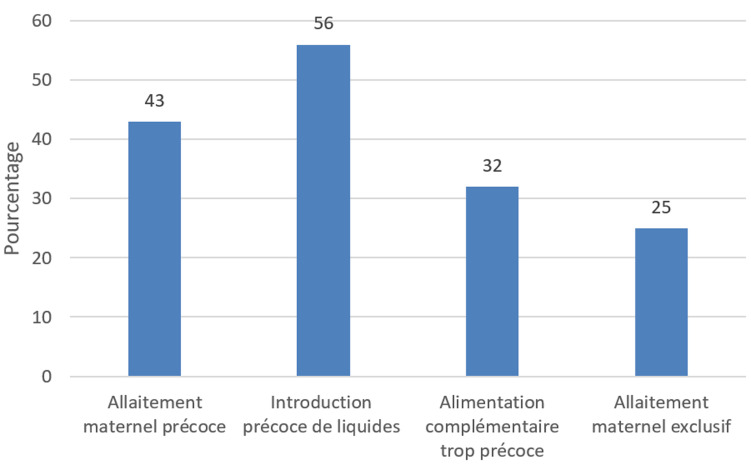
pratique des mères d'enfant de 0-24 mois sur l'alimentation infantile

Nous avons mené une étude transversale descriptive du 1^er^ au 30 juin 2022 dans la sous-préfecture de Sibut, auprès de 413 mères d'enfants âgés de 0 à 24 mois. Les résultats montrent que 65,6% des mères reconnaissent les bienfaits du colostrum qui est le premier lait sécrété après l'accouchement, 59,8% connaissent l'allaitement exclusif, et 65,4% estiment que les aliments complémentaires dès six mois sont bénéfiques. Toutefois, des idées erronées persistent: 72,9% pensent que le lait de vache est équivalent au lait maternel, et 49,4% croient que l'eau peut être donnée avant six mois (Tableau 1). Concernant les pratiques, bien que toutes les mères aient allaité, seulement 43% ont initié l'allaitement dans l'heure suivant la naissance. Par ailleurs, 56% des enfants de moins de six mois ont reçu des liquides autres que le lait maternel, principalement de l'eau. En matière d'alimentation complémentaire, 32% des nourrissons ont reçu des aliments avant trois mois, et seulement 25% étaient encore sous allaitement exclusif au moment de l'enquête. La fréquence des repas solides après un an reste également insuffisante (Figure 1).

**Tableau 1:** connaissances des mères d'enfant de 0-24 mois en matière d'alimentation infantile

Questions	% Réponses correctes	% Réponses incorrectes	% Reponses incertaines
**Connaissance sur allaitement maternel**			
Donner du colostrum est bon pour la santé du nouveau-né	65.6%	24.0%	10.2%
Le lait maternel est suffisant jusqu’à 6 mois	59.8%	28.8%	11.4%
Allaiter l’enfant après 1 an peut donner la malnutrition	28.1%	58.8%	13.1%
Le lait de vache a la même qualité que le lait maternel	72.9%	17.7%	9.4%
Un bébé allaité malade doit téter plus souvent	40.8%	53.9%	5.3%
**Connaissance sur aliment de complément**			
Donner des aliments semi-liquides et solides dès 6 mois	65.4%	24.9%	9.7%
Au moins 4 repas par jour après 6 mois	34.4%	42.2%	9.4%
Alimentation complémentaire doit inclure bouillie, légumes, fruits, céréales	63.4%	24.2%	12.4%
L’eau peut être donnée avant 6 mois	49.4%	43.1%	7.5%
Aliments mixtes peuvent être introduits progressivement	52.1%	33.4%	14.5%
Une mauvaise hygiène alimentaire peut rendre les enfants malades	67.1%	25.7%	7.2%

Ces résultats soulignent la nécessité de renforcer les interventions communautaires en matière d'éducation nutritionnelle, qui constitue un levier essentiel pour améliorer les connaissances et les pratiques des mères. En effet, l'éducation nutritionnelle permet non seulement de promouvoir des comportements alimentaires sains, mais aussi de prévenir les formes les plus graves de malnutrition telles que le retard de croissance et l'émaciation. Selon la FAO, l'accès à une éducation nutritionnelle de qualité est vital pour permettre aux individus d'atteindre leur plein potentiel physique et cognitif, et constitue un droit fondamental lié à la sécurité alimentaire [8]. De son côté, l'UNICEF souligne que les programmes de sensibilisation et de conseil nutritionnel auprès des familles sont indispensables pour garantir une alimentation adéquate dès la petite enfance, améliorer les pratiques d'allaitement et d'alimentation complémentaire, et ainsi réduire les risques de malnutrition aiguë et chronique [9,10].

**Conclusion:** les résultats de cette étude révèlent des insuffisances notables dans les connaissances et les pratiques nutritionnelles des mères à Sibut, malgré une certaine sensibilisation aux recommandations de l'OMS. Ces lacunes contribuent à maintenir une situation nutritionnelle préoccupante chez les enfants de moins de cinq ans. Il est donc impératif de renforcer les interventions communautaires en matière d'éducation nutritionnelle, en s'appuyant sur des stratégies adaptées au contexte local. L'éducation nutritionnelle, reconnue par la FAO et l'UNICEF comme un outil fondamental de prévention, peut améliorer durablement les comportements alimentaires et contribuer à la réduction de la malnutrition infantile en République centrafricaine.

## Remerciements

Nous adressons nos sincères remerciements à Monsieur le Ministre de la Santé et de la Population pour nous avoir accordé l'autorisation de collecte des données dans le cadre de cette étude. Nous exprimons également notre gratitude à l'équipe cadre du district sanitaire de Kémo ainsi qu'au personnel des formations sanitaires sélectionnées, pour leur collaboration et leur disponibilité tout au long de la réalisation de cette enquête. Nos remerciements vont aussi aux enquêteurs, dont l'engagement et le sérieux ont grandement contribué à la qualité des données collectées. Enfin, nous remercions chaleureusement les mères d'enfants qui ont accepté de participer à cette étude et de partager leurs expériences avec bienveillance.
